# Biomedical research in Nigeria: realities and misconceptions

**DOI:** 10.2144/fsoa-2019-0157

**Published:** 2020-04-15

**Authors:** Bashiru Garba, Bashir Saidu

**Affiliations:** 1 Department of Veterinary Public Health & Preventive Medicine, Faculty of Veterinary Medicine, Usmanu Danfodiyo University, Sultan Abubakar Road, City Campus Complex, Sokoto 840212, Nigeria; 2 Department of Veterinary Physiology & Biochemistry, Faculty of Veterinary Medicine, Usmanu Danfodiyo University, Sultan Abubakar Road, City Campus Complex, Sokoto 840212, Nigeria

**Keywords:** biomedical research, human capital development, innovation, international funding, low-income countries, misconceptions, Nigeria, research collaboration, research impact, research institutes

## Abstract

Investment in biomedical research is believed to drive economic growth and increase human capital, leading to increased productivity and sustainability. Unfortunately, such positive impacts are not palpable among the resource-poor countries. This can be attributed to the poor quality of research findings and the reliability of findings, which often are rarely translated to impactful products or decisions. While the Nigerian governments are making considerable efforts to improve the quality of research through increased funding, as well as sponsorship and training of scholars in technologically advanced institutions. This is in order for the transfer of knowledge to improve the livelihood of its citizens. However, there is still need for the private multinational organizations to support this course.

## Lay abstract

Lack of quality research constitutes an impediment to the growth and development in Nigeria. Despite the enormous human resources Nigeria has, only a fraction of its research outputs is palpable. This is despite the challenges facing the country, many of which are manageable via robust and efficient research. Notably, among these barriers are lack of funding, equipment and mentoring. Addressing these barriers is a step toward improving research capacity and output. This paper represents a perspective on ways to tackle the challenges as well as the success stories recorded.

Research as a whole is about satisfying one’s curiosity, about identifying a problem and looking for ways to solve them. It varies depending on context. In other words, it could be biomedical, agricultural, technological or even in the humanities [1]. In this article, emphasis is made on biomedical research. Research is a reflective endeavor, it is equally considered an intellectual investigation aimed at discerning, deducing and reviewing human empirical, factual and conceptual knowledge [2]. ‘Biomedical research’ encompasses a series of activities traversing multiple disciplines in biology and medicine [1]. Within the scope of these disciplines are experiments designed to investigate and understand reality. This is done by studying events at different stages, from microscopic, molecular and cellular levels, to the organism and the population levels. However, the term ‘biomedical research’ can have a much broader scope depending on professional organizations or academic departments [3]. In medical and health sciences, biomedical research is considered a broad area of science, which involves the investigation of the biological process and the causes of disease. This is achieved via careful experimentation (controlled and uncontrolled), observation, laboratory work, analysis and testing [1]. Essentially, what it seeks to do is to search for knowledge, for truth, for solutions to problems and make efforts to prove a hypothesis right or wrong. However, to do that, a problem must be identified, relevant pieces of literature must be consulted to propose possible solutions, data will need to be generated, analyzed and an outcome (inference) deduced.

## Biomedical research in resource-poor countries

Considerable differences in the economy and livelihood of industrialized nations and economically constrained countries constitute a serious threat to global health research. This disparity also presents ethical challenges concerning the quality of research outcomes from these regions. In recent years, the migration of people from these resource-limited countries driven by conflicts, wars, insurgencies, and the search for greener pastures has come with undesirable effects. As a result, a significant amount of health problems and disease threats seem to be crossing international borders at a global scale. The emergence of many infectious diseases and the threat they pose to public health are further sustained by increasing global commerce, travel and disruption of ecological systems [4]. Hence, developed nations are compelled to pay attention by providing increased funding for healthcare delivery and the execution of problem-solving research in order to improve the quality of life and increase food supply, so as to stem the tide of problematic emigration [5]. For the most part, these interventions are not only to address the threats of disease transmission, rather, these clinical researches sponsored by rich academic institutions and pharmaceutical companies are being exploited for the benefit of patients in developed countries [6]. Other factors that pose significant challenges to biomedical research in resource-poor settings include; communication gaps that affect the comprehension of information, conflict of interest concerning the incompatible nature of recognized ethical standards for research and, the social, political and economic inequities in these developing countries [7]. As a result, vulnerability and exploitation by multinational organizations, the WHO has put forward some recommendations that will ensure ethical practices and returns benefits to the study community. These recommendations include; respect for the traditional norms and values of the study communities; encouraging collaborative partnerships; upholding ethical standards in research and provision of feedback to the study participants and community. Observing these recommendations will go a long way in closing the wide gaps knowledge between these two research entities.

## Status of research in Nigeria

In developed nations, opportunities for researchers and other investigators to undertake innovative research are plentiful. This has resulted in the researchers acquiring the enhanced ability for collaboration within institutions and with others around the world [8]. In these technologically advanced nations, the consistent advances in the area of science and technology have allowed such interactions to blossom rapidly. Nigeria is characterized by a diverse ethnic, religious and political structure. Worthy of note is the fact that research is mostly undertaken by government-owned research institutes and other institutes of higher learning (universities, colleges of agriculture, etc.). Traditionally, the establishment and operation of these public institutions (research institutes, universities, monotechnics and polytechnics) reflects the socioeconomic and political diversity of the country. Nigeria has the largest human population in Africa and consequently, the largest number of students enrolled in the higher education sector [7]. Productivity in research is a quintessential indicator of efficiency in any production system. Ironically, research productivity is measured as the number of publications per researcher in institutions such as the universities, or research institutes [9]. Recent investigations of the research productivity among six selected West African countries revealed that there had been a significant increase in scientific publications in Nigeria compared with the other countries under consideration [9].

However, do these high number of publications correspond to the rate of citations, impact on communities, advancement in medical and veterinary care; or even improvements in life as is the case in other climes? The impression that publication output equates to research productivity is an erroneous one. Assessment of impact generated as a result of research varies depending on whether it is an ‘academic impact,’ which is the intellectual contribution within the academic circle, ‘external socioeconomic impact,’ or both [10]. A better assessment would be to what extent do these research activities make a real difference of some sort in the livelihood of the study population? This is fundamentally what is meant by saying your research has an impact. Nonetheless, knowledge generated and publications leading to new products and services are equally viewed as impacts [11]. Realizing this fact may be behind the motivation of the present Federal Government of Nigeria’s increased funding for research and training, believing that these researches will produce positive impacts on the scientific, technological and socioeconomic development of the country. It is thought that these commitments will lead to economic growth, increase human capital and the development of pro-poor products and technologies, as well as provide evidence to inform policies and practice. However, are these aspirations realizable with the present researches conducted in our universities and research institutes?

It is intriguing to note that academics conduct the majority of the research in life science. This is borne out of the fact that publication is a prerequisite for the progression of an individual academic as well as a medium used to judge the contribution and ranking of their respective institutions. Recognition by the scientific community is granted to an academician or researcher only when their works are published. Similarly, their progression is tied to the number and quality of their publications, including promotions, research funding, travel grants and scholarships [12]. It is so demanding that an academician can stagnate or be demoted if they fail to produce the required number of publications during their waiting period as required by the university commission, giving rise to the notion of ‘force to publish or perish’ which often leads to many desperate and novice academicians into exaggerating their research findings and some publishing in fake nonpeer reviewed journals [12].

It is widely acknowledged that Nigerians are industrious, innovative, inquisitive and rich in patience and perseverance. These are all attributes of good researchers. In other words, Nigerians make excellent researchers and have proven to do so globally. Outside the shores of the country, Nigerians do very well. A lot of the research proposals that require grants/funds by many institutions, for instance in Southeast Asia (personal view), are developed by Nigerian postgraduate students. Unfortunately, lack of funding, lack of equipment and materials, as well as lack of awareness have all contributed to these potentials not being explored. Amid all the challenges mentioned above, an academic is expected to train students (undergraduate and postgraduate), to write books and to create technologies that can help prevent diseases and improve the productivity of animals etc. These are some of the main contributing factors that drive researchers into cheating and publishing false and inflated results. Yes, to a large extent, many results and claims about researches conducted in Nigeria today are exaggerated, however, it will be unfair not to acknowledge the genuine efforts of some researchers concerning the information they publish. It is also important to note that a large chunk of researches in Nigerian Universities and other institutions of higher learning are conducted during postgraduate training, which to a large extent are personally financed by the postgraduate students themselves with occasional support from some tutors [13,14]. In these circumstances, there can be no room for error. In other words, while it is common to conduct a study and not get the expected results, one will be required to repeat and repeat; in the above scenario, it will be extremely difficult. Meaning, the result must come out by any means necessary. With such plentiful obstacles, it is challenging for an individual to finance any meaningful research up to the point of conclusion. So yes, there is so much accurate information concerning published research outcomes in Nigeria, but at the same time, many other claims are misrepresentation of facts.

## Prospects

The need for a highly qualified workforce at the leading edge of research institutions and institutions of higher learning is critical for the development of our country. It is believed that the economic development and opportunities for a country’s citizen depend mainly on the strengths and successes of research universities [15]. Economic growth among top nations globally is driven by knowledge. In these types of knowledge-based economies, development is dependent on the amount of investments in education, learning and training. It is gradually becoming obvious that universities have central roles to play in the construction of knowledge-based economies. In other words, prosperous countries will be those that nurture and promote their research universities [15]. There is an urgent need for skills in resource-poor countries, which can be acquired through involvement in research. These skills can be developed through higher education, as well as capacity-building programs. Availability of funds dramatically influences the quality and impact of research and this is an essential requirement for any research institution. Unfortunately, the majority of the research funding in Nigeria is sponsored by the government, except for a handful of some international funding agencies [16]. Engaging in a public–private partnership has proven to be successful in generating funding for research, as well as incentivizing the development of new products and technologies.

Secondly, the need for collaborations is equally essential. Collaboration in the scientific research world is a hot buzzword. Working with individuals with different ideas and perspectives as well as different areas of expertise, can only result in better ideas and outcomes. Promoting effective research collaboration between the universities, research institutes and industries (both national and international) is lacking. Such collaborations will help in pushing the frontiers of knowledge and development of new technologies, thereby making them powerful engines for innovation and economic growth [17]. Academic and research collaborations are valuable tools that, in addition to accelerating the progress of an institution, enhances the quality of the work and extends the repertoire of the partners. This approach to research is beneficial to the faculty in learning and acquiring new knowledge and skills, thereby increasing the breadth of their knowledge and learning different approaches to solving a problem. It has proven to be successful in developed countries; however, in Nigeria, it has been largely neglected. This is partly because some researchers prefer working on their own. Although, this can be simply due to differences in personalities but much of the time it is because some people don’t like sharing credits while others aren’t good team players. Therefore, there is an urgent need for a reorientation and change of approach in order to give room and accommodate this strategy for the overall benefit of the nation. In this regard, both intra- and inter-institutional collaboration must be emphasized, encouraged and instilled to a level where it can impact and improve the quality, resources and capabilities of both the researchers and institutions involved.

Furthermore, every serious nation must invest in its human capital and development. Human capital can only be developed via research. Nigeria’s socioeconomic performance and rating in human development indices, which are usually a reflection of its human capital status, are low [18]. In this vein, research can be viewed as a tool for enhancing human capital development. It is believed that addressing the following issues will go a long way in mitigating the problems researchers face in Nigeria. Moreover, improved funding to research institutions and rewarding exceptional breakthrough, education and training for health professionals as well as creating awareness are all important and necessary steps toward the appreciation for the value of research. Nonetheless, the government is making efforts to address the issue of human capital development by the introduction of the Tertiary Education Trust Fund (TETFund) scheme, in addition to the existing National Science and Technology Fund [19]. The funds provide scholarships for academics to pursue Postgraduate degree programs in more scientifically advanced countries. The scheme is set out to propose and undertake research as well as create reliable databank for improvement of education in Nigeria [20]. It is also involved in the recruitment, training and retraining of a highly motivated workforce [20]. The next stage should be to empower these (trained) individuals with grants and provide enabling environment for the conduct of researches to address peculiar challenges bedeviling our nation.

## Misconceptions

The role of research is to provide a platform for obtaining answers to questions by studying evidence using the scientific methods [21]. In Nigeria, the majority of the time, research efforts terminate at the awarding of postgraduate degrees. However, in other instances, it proceeds to industrial application.

Similarly, researches conducted in the area of biomedical sciences are predominantly repetitive. Not that it is wrong to conduct replicative studies, but it lacks originality and it doesn’t give room for novel discoveries in most cases, especially in the face of peculiarities in Nigeria, this often influences research outcomes such as climate, livelihood, disease endemicity, etc. [22]. From the perspective of an individual research career, replicative studies are also fraught with drawbacks, including reluctance to publish such works by reputable high impact journals since it doesn’t qualify as new research, therefore is of less interest to readers. Unfortunately, lack of originality, novelty or significance often lead to rejection by most of the prestigious journals, hence the less likely it is that the study will receive funding.

A critical point to also note concerning quality research in Nigeria is that, a multitude of outstanding research is resting in libraries in the form of thesis’ and dissertations’ without being published [9]. Except for those in academia, in Nigeria, publication is not much of a requirement for most career progression. Meanwhile in developed nations, publications in high impact journals are a compulsory requirement for graduation. This has hampered the image of Nigerian researchers and research institutions among their peers as successful publication of research brings attention to scholars and their institutions [23]. Recent studies have reported lack of funding, lack of research equipment and facilities, and inadequate training as some of the barriers that affect the conduct and publications of research findings among young researchers [14].

Finally, mentorship is an integral part of developing into a successful researcher. It is linked to enhanced productivity, and self-efficacy of the mentee [24]. Unfortunately, studies have indicated that trainees from disadvantaged groups, particularly in Africa, receive less mentoring [24].

## Conclusion & future perspective

The benefits of research activities to human capital development and economic stability are huge. As observed, reports indicate that research can bring about positive economic returns. It is equally a proven fact that knowledge generated from public investment in research can aid policy and practice decisions. Unfortunately, the capacity to access and use research among low-income countries is low. However, unethical practices and misconducts driven by lack of funding and support is a cause for concern in modern biomedical research. Finally, if politics and favoritism are shunned in the selection of research proposals for award, if adequate publicity during call for research grants applications is ensured, if more awareness and orientation on international funding agencies is provided, many of the factors that hinder our progress in the area of research in Nigeria would be solved. Hence, concerted efforts to redeem the image and integrity of biomedical research in Nigeria and its reportage is desired more than ever.

**Executive summary**

- Nigerian universities which are the centers for knowledge, research and innovation often do not attain the greatness they deserve.
- Research in Nigeria is yet to impact the socioeconomic well-being of its citizens.
- There is a need for other multinational organizations to support research efforts is desired.
- Interdisciplinary collaboration enhances the capacity of researchers. Mentorship should also be promoted.

## References

[1] Flier JS, Loscalzo J Categorizing biomedical research: the basics of translation. FASEB J. 31, 3210–3215 (2017). 28765169 10.1096/fj.201700303R PMC5503706

[2] Fang FC, Casadevall A Lost in translation – basic science in the era of translational research. Infect. Immun. 78(2), 563–566 (2010). 20038540 10.1128/IAI.01318-09 PMC2812192

[3] Zerhouni EA US biomedical research: basic, translational, and clinical sciences. JAMA 294(11), 1352–1358 (2005). 16174693 10.1001/jama.294.11.1352

[4] Vignier N, Bouchaud O Travel, migration and emerging infectious diseases. EJIFCC 29(3), 175–179 (2018). 30479600 PMC6247124

[5] De Maeseneer J, Van Weel C, Egilman D Funding for primary health care in developing countries: money from disease specific projects could be used to strengthen primary care. BMJ. 336(7643), 518–519 (2008). 18325939 10.1136/bmj.39496.444271.80 PMC2265338

[6] Weigmann K The ethics of global clinical trials. EMBO Rep. 16, 566–570 (2015). 25851646 10.15252/embr.201540398 PMC4428044

[7] Akudolu L-R, Adeyemo KS, Jowi J Research and PhD capacities in Sub-Saharan Africa: Nigeria Report British Council & German Academic Exchange Service, UK (2018).

[8] Holbrook KA, Sanberg PR Understanding the high cost of success in university research. Technol. Innov. 1(3), 269–280 (2013). 10.3727/194982413X13790020922068 PMC3987954 24744822

[9] Odeyemi OA, Odeyemi OA, Bamidele FA, Adebisi OA Increased research productivity in Nigeria: more to be done. Futur. Sci. OA 5(2), FSO360 (2019). 10.4155/fsoa-2018-0083 PMC6391628 30820343

[10] Penfield T, Baker MJ, Scoble R, Wykes MC Assessment, evaluations, and definitions of research impact: a review. Res. Eval. 23(1), 21–32 (2013).

[11] Duryea M, Hochman M, Parfitt A Measuring the impact of research. Research Global 1, 8–9 (2007).

[12] Samuel A, Aranha V Valuable research in fake journals and self-boasting with fake metrics. J. Pediatr. Neurosci. 13(4), 517–518 (2018). 30937105 10.4103/JPN.JPN_66_18 PMC6413586

[13] Desmennu AT, Owoaje ET Challenges of research conduct among postgraduate research students in an African University. Educ. Res. Rev. 13, 336–342 (2018).

[14] Okoduwa SIR, Abe JO, Samuel BI Attitudes, perceptions, and barriers to research and publishing among research and teaching staff in a Nigerian Research Institute. Front. Res. Metrics. Anal. 3, 26 (2018).

[15] Arai K, Cech T, Chameau JL The future of research universities. Is the model of research-intensive universities still valid at the beginning of the twenty-first century? EMBO Rep. 8(9), 804–810 (2007). 17767189 10.1038/sj.embor.7401052 PMC1973958

[16] Baro EE, Bosah GE, Obi IC Research funding opportunities and challenges: a survey of academic staff members in Nigerian tertiary institutions. Bottom Line 30, 47–64 (2017).

[17] Falode OA, Nebeife PC Promoting effective university-oil industry research collaboration in Nigeria. Soc. Pet. Eng. – 37th Niger. Annu. Int. Conf. Exhib. NAICE 2013 - To Grow Africa's Oil Gas Prod. Required Policy, Funding, Technol., Tech. Capab. 1, 510–521 (2013).

[18] Chikwe CK, Ogidi RC, Nwachukwu K Challenges of research and human capital development. J. Edu. Practice. 6(28), 44–47 (2015).

[19] Oluwasanu MM, Atara N, Balogun W Causes and remedies for low research productivity among postgraduate scholars and early career researchers on non-communicable diseases in Nigeria. BMC Res. Notes 12, 403 (2019). 31307552 10.1186/s13104-019-4458-y PMC6628473

[20] Bogoro Suleiman Elias Synopsis of my vision for TETFUND (2014). www.tetfund.gov.ng/index.php/2-uncategorised/4-our-vision

[21] Venkataram P An article to clear up some misconceptions about the nature of research. (2012). https://nitt.edu/home/academics/departments/physics/programmes/phd/motivation_principles-1.pdf

[22] Kumwenda S, Niang EHA, Orondo PW Challenges facing young African scientists in their research careers: a qualitative exploratory study. Malawi. Med. J. 29(1), 1–4 (2017). 28567188 10.4314/mmj.v29i1.1 PMC5442483

[23] Rawat S, Meena S Publish or perish: where are we heading? J. Res. Med. Sci. 19(2), 87–89 (2014). 24778659 PMC3999612

[24] Sorkness CA, Pfund C, Ofili EO A new approach to mentoring for research careers: The National Research Mentoring Network. BMC Proc. 11, 22 (2017). 29375663 10.1186/s12919-017-0083-8 PMC5773914

