# Corrigendum

**DOI:** 10.2144/fsoa-2019-0157c

**Published:** 2024-05-16

The research article by authors Bashiru Garba & Bashir Saidu which appeared in the April 2020 issue of *Future Science OA* 6(7), FSO475 (2020), was published with the author's name being presented incorrectly as ‘Bashir Sa'idu’. This has now been corrected to ‘Bashir Saidu’.

The editors and the authors of *Future Science OA* would like to sincerely apologize for any inconvenience or confusion this may have caused our readers.

